# Correction to: An Erwinia amylovora inducible promoter for improvement of apple fire blight resistance

**DOI:** 10.1007/s00299-022-02887-6

**Published:** 2022-06-08

**Authors:** Matthieu Gaucher, Laura Righetti, Sébastien Aubourg, Thomas Dugé de Bernonville, Marie-Noëlle Brisset, Elisabeth Chevreau, Emilie Vergne

**Affiliations:** 1 Univ Angers, Institut Agro, INRAE, IRHS, SFR QUASAV, 49000 Angers, France; 2 Research Centre for Cereal and Industrial Crops (CREA-CI), Council for Agricultural Research and Agricultural Economics Analysis, Via di Corticella 133, 40128 Bologna, Italy; 3 EA2106 Biomolécules et Biotechnologies Végétales, UFR Sciences Pharmaceutiques, Université François Rabelais, 31 avenue Monge, 37200 Tours, France

## Correction to: Plant Cell Reports 10.1007/s00299-022-02869-8

The first and last name of all authors have been incorrectly swapped in the original publication. The complete correct names of all authors should read as follows.

Matthieu Gaucher

Laura Righetti

Sébastien Aubourg

Thomas Dugé de Bernonville

Marie‑Noelle Brisset

Elisabeth Chevreau

Emilie Vergne

The original article has been corrected.

