# Supplementary material for: An Erwinia amylovora inducible promoter for improvement of apple fire blight resistance
Source: Plant Cell Rep. 2022 Apr 6;41(7):1499–513. doi: 10.1007/s00299-022-02869-8 (PMC9270298; doi:10.1007/s00299-022-02869-8)
Supplement: Supplementary file 1 — Supplementary file1 (PDF 398 kb) [file 299_2022_2869_MOESM1_ESM.pdf]

An *Erwinia amylovora* inducible promoter for improvement of apple fire blight resistance

Gaucher Matthieu[1], Righetti Laura[1], Aubourg Sébastien[1], Dugé de Bernonville Thomas[2], Brisset Marie-Noëlle[1], Chevreau Elisabeth[1], Vergne Emilie[1]

[1] Univ Angers, Institut Agro, INRAE, IRHS, SFR QUASAV, F-49000 Angers, France
[2] EA2106 Biomolécules et Biotechnologies Végétales, UFR Sciences Pharmaceutiques, Université François Rabelais, 31 avenue Monge, 37200 Tours, France

Matthieu Gaucher and Laura Righetti should be considered joint first authors

Correspondence: Emilie Vergne
ORCID ID : 0000-0001-9280-8688
Emilie.vergne@inrae.fr

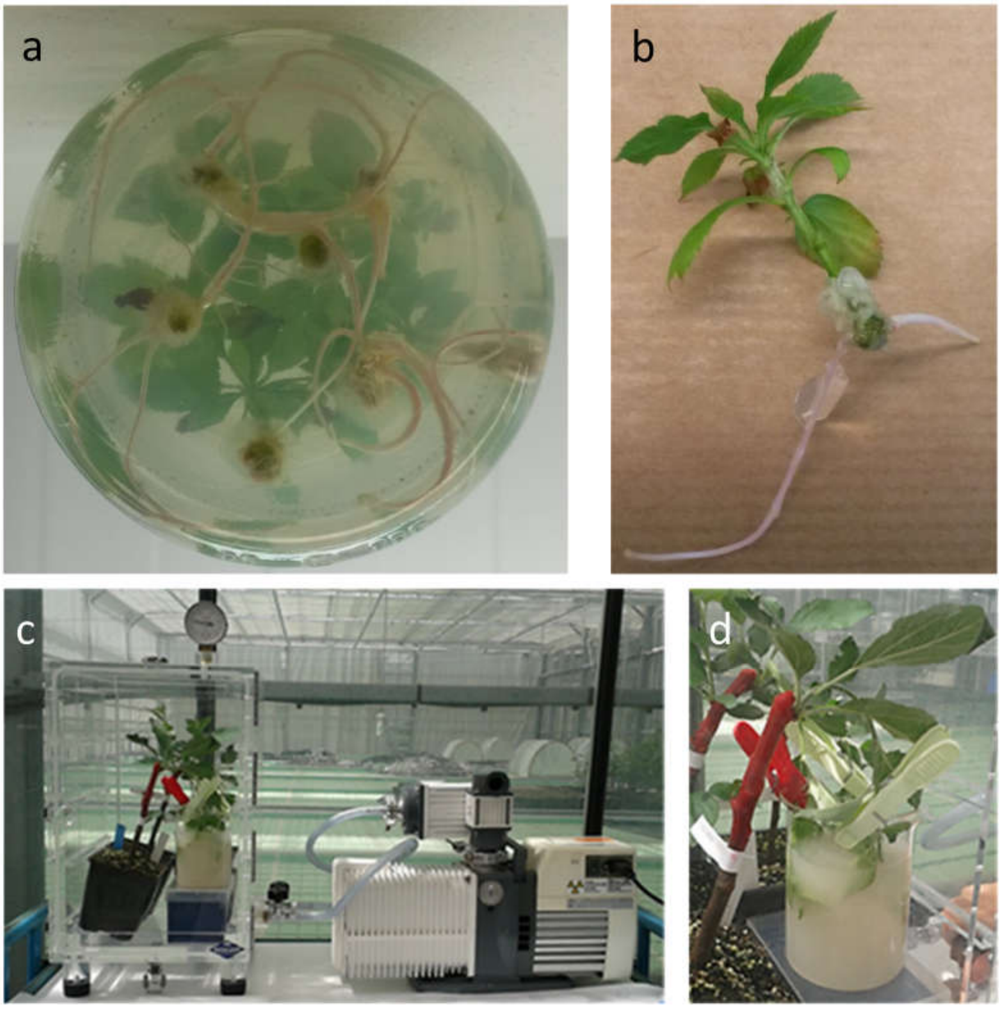

Online resource 1 Pictures of some materials and methods. a) & b) "*In-vitro*" growing shoots 4 weeks after rooting. c) Vacuum chamber and pump used to infiltrate bacteria in leaves. d) Growing shoots of young grafts submerged in bacterial suspension to infiltrate bacteria in tissues by vacuum

Online resource 2 Agrobacterium strains used in this work

Strain	Vector	Resistance	Insert	Helper	Resistance	Use (concentration)
EHA105	pKGWFS7 (Karimi et al., 2002)	Spectinomycin 50 µg/mL	*p35S*<br>*pKFDV02* (MM106; 2030 bp)<br>*pPPO16* (MM106; 2219 bp)	pBBR-MCS5	Gentamicin 50 µg/mL	Transient or stable transformation (1×10^9 CFU/mL)
	pBin61 (Bendahmane et al., 2000)	Kanamycin 50 µg/mL	*p35S:p19* (Voinnet et al., 2003)	pBBR-MCS5	Gentamicin 50 µg/mL	Transient transformation (2.5×10^8 CFU/mL)
	pGREEN II 0800 LUC (Hellens et al., 2005)	Kanamycin 50 µg/mL	*p35S* (1027 bp)<br>*pKFDV02* (MM106 2030 bp)<br>*pPPO16* (MM106; 1177 bp)	pSoup	Tetracycline 10 µg/mL	Transient transformation (5×10^8 CFU/mL)

Online resource 3 Primers used in this work

**Gene/family name**	**Accessions/ references**	**Forward primer 5'-3'**	**Reverse primer 5'-3'**	**Analyses**
		PPO and PPO-like genes		
MdPPO16	MD10G1299400a	TGCCCGCCGCCTTCCAC	GCTCCATCGCTTTGTAGTATTTGTC	RT-qPCR
MdKFDV01	MD05G1319000a	TCCAAACAGGGTCGTCGAACT	TGCGCTTCCTTTTCCGCC	RT-qPCR
MdKFDV02	MD10G1298200a	CGTGCATGTGAACGATGATGAG	AACTCCCAAGTCCTCCAACAA	RT-qPCR
		Promoters		
p35S	from pK7WG2D from Karimi et al. (2002)	CACCACTAGAGCCAAGCTGATCTC	TCGACTAGAATAGTAAATTGTAAT	Cloning in pKGWFS7 via a pENTR/SD/D-TOPO
		CACCGGTACCACTAGAGCCAAGCTGATCTC (KpnI)	CCCAAGCTTTCGACTAGAATAGTAAATTGTAAT (HindIII)	cloning in pGREEN II 0800 LUC

pPPO16	MK873007	CACCGTTTCTTCATCCTGCTGCTT	GGCTTAGCTCTCTGGTTTTG	Cloning in pKGWFS7 via a pENTR/SD/D-TOPO
		AAGGTACCTATCTGGCCAATTGCCTTGT (KpnI)	AACCATGGCTTAGCTCTCTGGTTTTG (NcoI)	cloning in pGREEN II 0800 LUC
pKFDV02	MK873006	CACCGGTACCGAAGCTCAAGAAAACTGG (KpnI)	GGTTTTTGTTTCCTTTTTGTCAAG	Cloning in pKGWFS7 via a pENTR/SD/D-TOPO
		CACCGGTACCGAAGCTCAAGAAAACTGG (KpnI)	AACCATGGTTTTTGTTTCCTTTTTGTCAAG (NcoI)	cloning in pGREEN II 0800 LUC
		Reporter genes		
REN (Renilla)	EU048863.1 Cloning vector pGreenII 0800 LUC	ATCGGACCCAGGATTCTTTT	ACTCGCTCAACGAACGATTT	RT-qPCR
FIRE (Firefly)	EU048863.1 Cloning vector pGreenII 0800 LUC	CCAGGGATTTCAGTC	AATCTCACGCAGGCAGTTCT	
GUS	from pKGWFS7 from Karimi et al. (2002)	GCACGGGAATATTTCGCCAC	ATAACGGTTCAGGCACAGCA	specific RT (reverse) and RT-qPCR
SPEC	from pKGWFS7 from Karimi et al. (2002)	ATCATTCCGTGGCGTTATCC	GCTGGACCTACCAAGGCAAC	
		Reference genes		
GADPH (Glyceraldehyde-3-phosphate dehydrogenase)	CN494000	GCTGCCAAGGCTGTTGGAA	CAGTCAGGTCAACAACGGAAAC	RT-qPCR
TuA (Tubulin alpha-1)	CO065788	GTTCAATGCTGTTGGTGGTG	CTGCGGAGAAGGATAGATGG	
ACTIN(Actin 7)	CV151413	CAACCTCTCGTCTGTGATAATG	GCATCCTTCTGTCCCATCC	
EF-1α (Elongation Factor)	AJ223969	CCTTCTTGAGGCTCTTGACCAG	CCAACAGGAACAGTACCGATACC	PCR
		Transgenic lines assesment		
AGRO	23S ribosomal RNA coding gene CP014260.1 gene locus_tag="AWN88_17620" 1310643..1313449	GTAAGAAGCGAACGCAGGGAACT	GACAATGACTGTTCTACGCGTAA	PCR
NptII	from plasmid pKGWFS7 from Karimi et al. (2002)	ATCGGGAGCGGCGATACCGTA	GAGGCTATTCGGCTATGACTG	
p35S:GUS	promoter from pK7WG2D and reporter gene from pKGWFS7 from Karimi et al. (2002)	CGCACAATCCCACTATCCTT	ACAGTTTTCGCGATCCAGAC	
pKFDV02:GUS	reporter gene from pKGWFS7 from Karimi et al. (2002)	AACCAATTGGGCTCGTGTAG	ACAGTTTTCGCGATCCAGAC	
pPPO16:GUS	reporter gene from pKGWFS7 from Karimi et al. (2002)	CTGCGGGGTATCTACATGGT	TAATGAGTGACCGCATCGAA	

a all PPO are available at https://iris.angers.inra.fr/gddh13, "curated CDS" layer

Online resource 4 Percent identity matrix of CDS and protein sequences of PPO in *Malus* x *domestica*

CDS		MdPPO02	MdPPO03	MdPPO05	MdPPO06	MdPPO08	MdPPO10	MdPPO12	MdPPO13	MdPPO15	MdPPO16
		**MD05G1319100**	**MD05G1319300**	**MD05G1319800**	**MD05G1320100**	**MD05G1320800**	**MD10G1298300**	**MD10G1298500**	**MD10G1298700**	**MD10G1299300**	**MD10G1299400**
**MdPPO02**	**MD05G1319100**	100	91,5	66,65	66,07	66,07	61,71	61,98	61,8	62,37	63,74
**MdPPO03**	**MD05G1319300**	91,5	100	66,99	66,13	66,3	61,77	61,8	61,63	62,31	64,77
**MdPPO05**	**MD05G1319800**	66,65	66,99	100	94,1	93,67	63,83	62,52	62,11	61,88	64,15
**MdPPO06**	**MD05G1320100**	66,07	66,13	94,1	100	91,45	64,13	63,22	62,52	62,52	64,8
**MdPPO08**	**MD05G1320800**	66,07	66,3	93,67	91,45	100	63,52	61,4	61,23	60,94	64,61
**MdPPO10**	**MD10G1298300**	61,71	61,77	63,83	64,13	63,52	100	59,87	60,05	59,93	61,22
**MdPPO12**	**MD10G1298500**	61,98	61,8	62,52	63,22	61,4	59,87	100	98,31	97,71	74,29
**MdPPO13**	**MD10G1298700**	61,8	61,63	62,11	62,52	61,23	60,05	98,31	100	97,71	74,01
**MdPPO15**	**MD10G1299300**	62,37	62,31	61,88	62,52	60,94	59,93	97,71	97,71	100	73,89
**MdPPO16**	**MD10G1299400**	63,74	64,77	64,15	64,8	64,61	61,22	74,29	74,01	73,89	100

Protein		MdPPO02	MdPPO03	MdPPO05	MdPPO06	MdPPO08	MdPPO10	MdPPO12	MdPPO13	MdPPO15	MdPPO16
		**MD05G1319100**	**MD05G1319300**	**MD05G1319800**	**MD05G1320100**	**MD05G1320800**	**MD10G1298300**	**MD10G1298500**	**MD10G1298700**	**MD10G1299300**	**MD10G1299400**
**MdPPO02**	**MD05G1319100**	100	92,49	61,14	60,62	60,66	51,58	54,25	54,08	54,93	56,55
**MdPPO03**	**MD05G1319300**	92,49	100	61,14	60,45	60,31	51,49	55,03	54,86	55,71	57,76
**MdPPO05**	**MD05G1319800**	61,14	61,14	100	93,36	91,79	54,77	54,43	53,74	52,52	57,17
**MdPPO06**	**MD05G1320100**	60,62	60,45	93,36	100	89,4	56,18	55,13	54,61	53,74	57,34
**MdPPO08**	**MD05G1320800**	60,66	60,31	91,79	89,4	100	56,03	54,01	53,66	52,61	56,39
**MdPPO10**	**MD10G1298300**	51,58	51,49	54,77	56,18	56,03	100	51,51	51,87	51,69	52,51
**MdPPO12**	**MD10G1298500**	54,25	55,03	54,43	55,13	54,01	51,51	100	97,54	96,23	70,97
**MdPPO13**	**MD10G1298700**	54,08	54,86	53,74	54,61	53,66	51,87	97,54	100	96,56	70,8
**MdPPO15**	**MD10G1299300**	54,93	55,71	52,52	53,74	52,61	51,69	96,23	96,56	100	70,63
**MdPPO16**	**MD10G1299400**	56,55	57,76	57,17	57,34	56,39	52,51	70,97	70,8	70,63	100

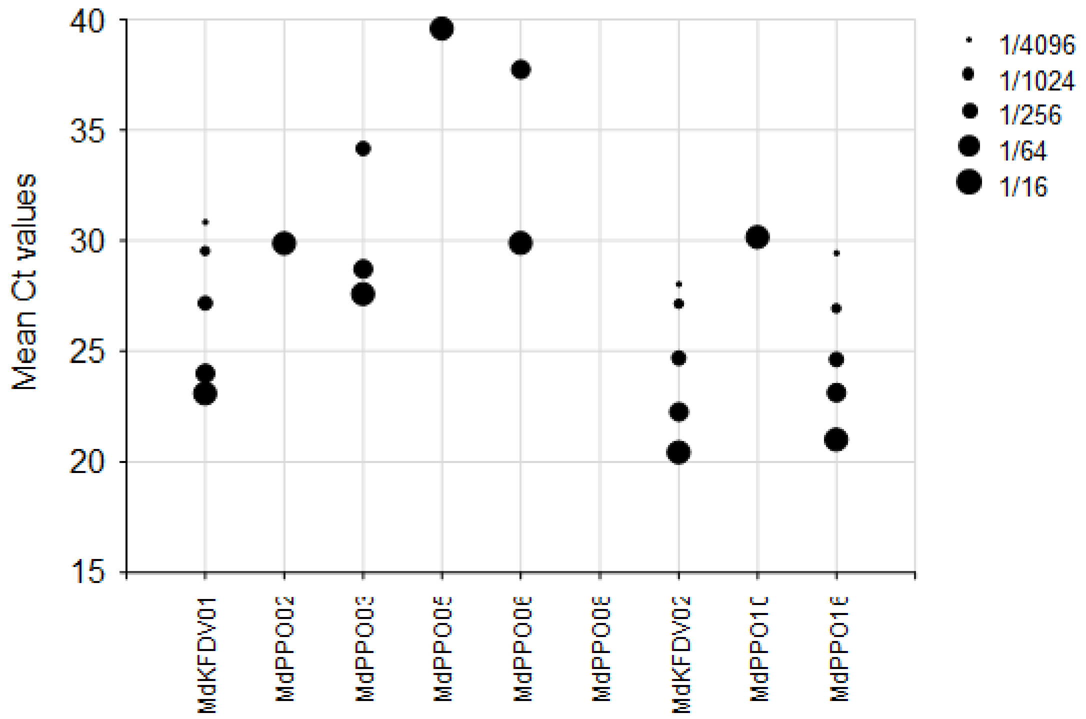

Online resource 5 Mean Ct values obtained by RT-qPCR with specific primers designed on each coding sequence (CDS) and tested using a 4-fold serial dilution (from 1/16 to 1/4096) of a cDNA pool (all samples of 'Evereste' and 'MM106'). Data were used to calculate primers efficiency and choose the genes for which the expression profiles were analyzed in the different samples (Fig. 2)

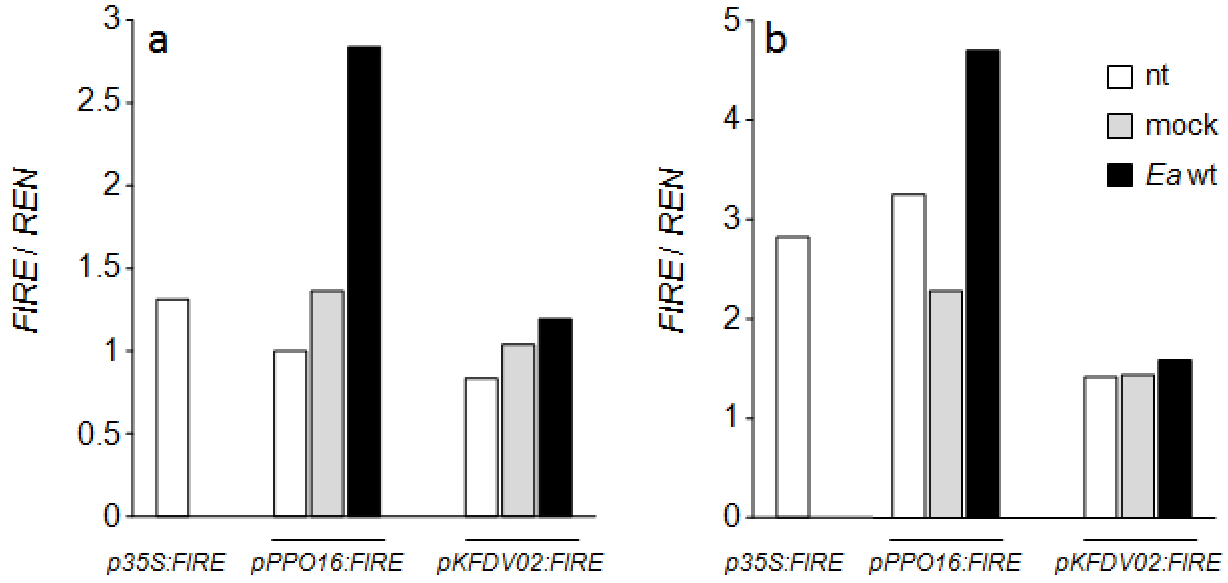

Online resource 6 Gene expression and activity of luciferase driven by *pPPO16* and *pKFDV02* in transient assays. Relative expression (A) and enzymatic activities (B) of firefly (FIRE) reporter driven by *p35S*, *pPPO16* and *pKFDV02* in untreated (nt, white), mock (light gray) or *Ea* wt (black) -infiltrated leaves (24 hpt) of transiently transformed 'Golden Delicious' *in vitro* plants, five days after agroinfiltration. *FIRE* raw expression levels (log2) of each sample were calibrated to the corresponding value of the sample *pPPO16:FIRE*-nt. Firefly luciferase expression and activity were normalized to Renilla luciferase (REN) expression and activity, respectively (n=1)

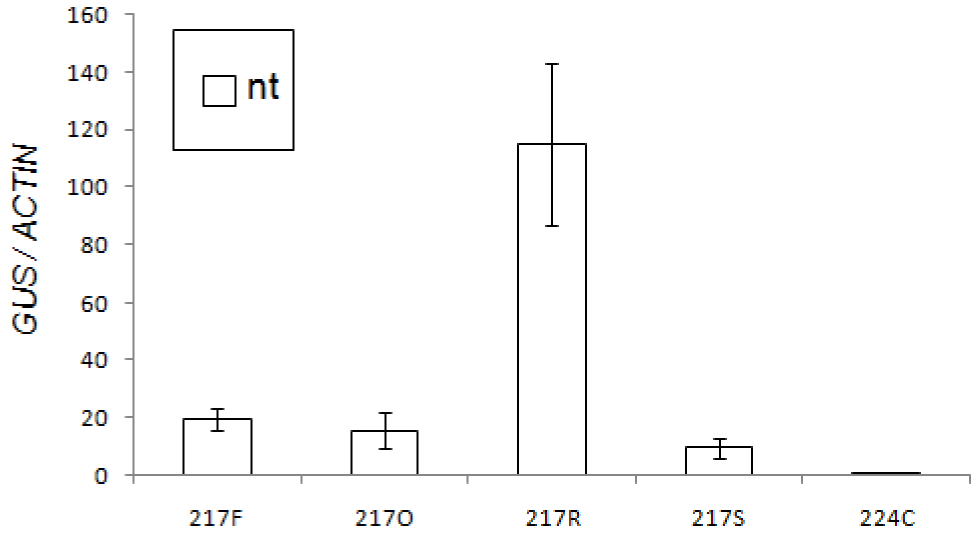

Online resource 7 *p35S*-driven *GUS* expression in four 'Golden Delicious' transgenic lines cultivated *in vitro*. Relative expression of *GUS* reporter gene driven by *p35S* in untreated leaves (nt) from transgenic lines 217F, O, R and S. *GUS* raw expression level of each sample are relative to the corresponding mean value in untreated leaves of the line 224C expressing *pPPO16:GUS*, and normalized with *ACTIN*. Bars represent SEM from 3 biological repeats (n=3). Lines 217O and S were kept for subsequent analyses

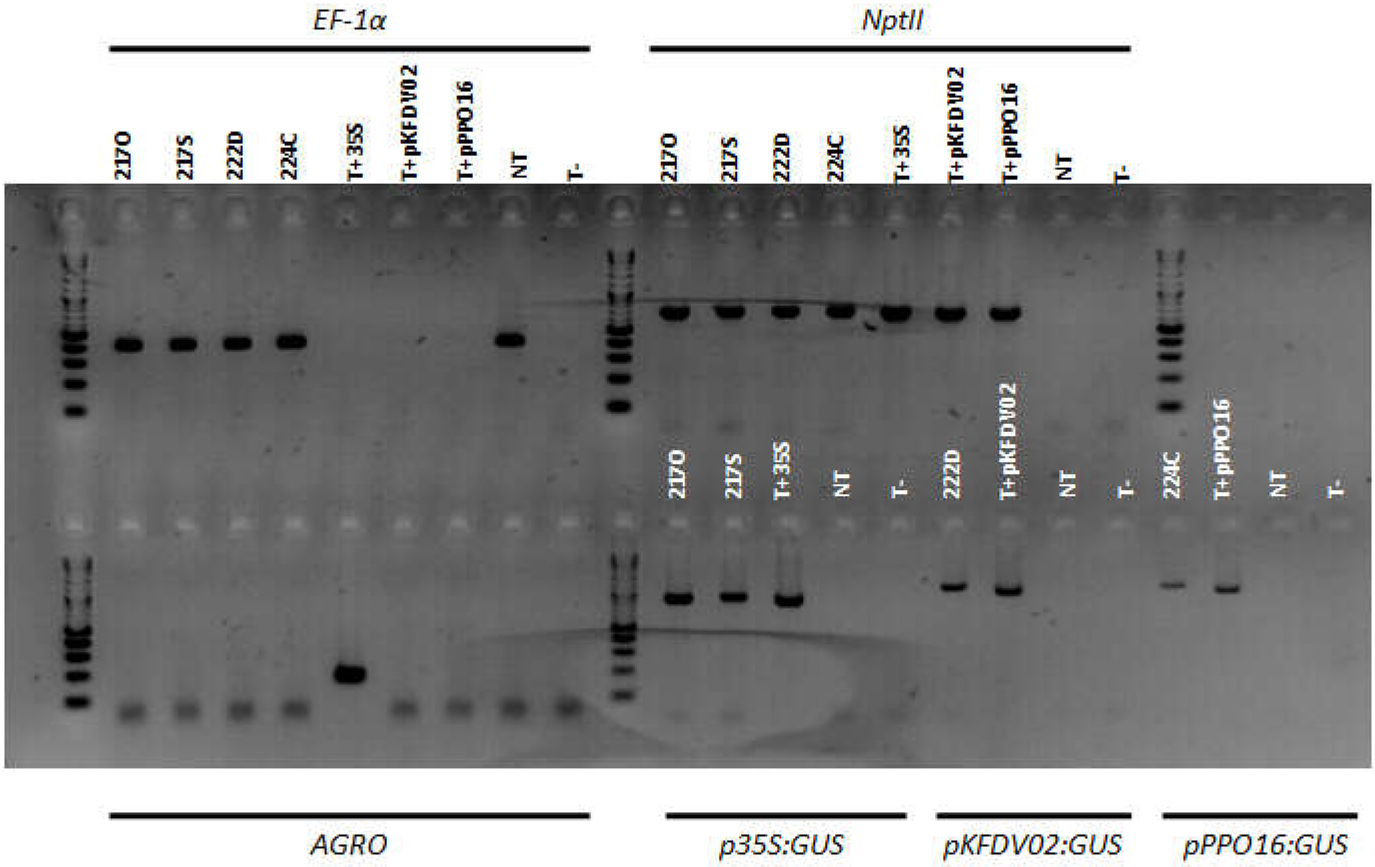

Online resource 8 Transgenic lines got are free from *A. tumefaciens* contamination. 217O & S: transgenic lines transformed with *p35S:GUS* construction, 222D: transgenic line transformed with *pKFDV02:GUS* construction, 224C: transgenic lines transformed with *pPPO16:GUS* construction, T+35S: DNA extraction of *A. tumefaciens* strain carrying pKGWFS7-*p35S:GUS* plasmid, as a positive control for transgenic lines transformed with *p35S:GUS* construction and *A. tumefaciens* presence, T+pKFDV02: DNA extraction of *E. Coli* strain carrying pKGWFS7-*pKFDV02:GUS* plasmid as a positive control for transgenic line transformed with *pKFDV02:GUS* construction , T+pPPO16: DNA extraction of *E. Coli* strain carrying pKGWFS7-*pPPO16:GUS* plasmid as a positive control for transgenic line transformed with *pPPO16:GUS* construction, NT: non-transformed 'Gala', T-:H20. *EF-1α*, *NptII*, *AGRO*, *p35S:GUS*, *pKFDV02:GUS*, *pPPO16:GUS*: primer couples

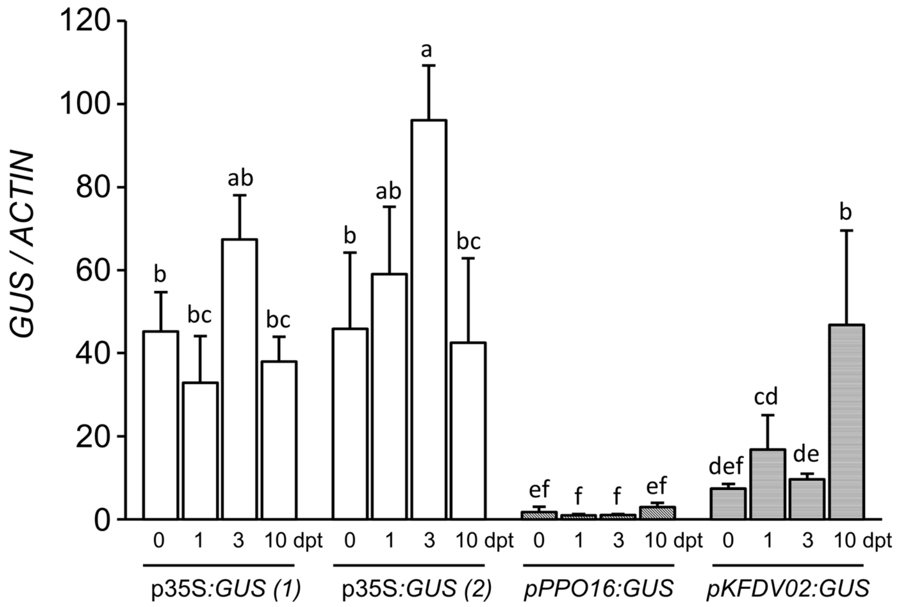

Online resource 9 Evolution of *GUS* expression over time in the youngest leaf of transgenic lines cultivated in greenhouse and challenged with *Vi*. Relative expression levels of *GUS* reporter gene promoted by *p35S*, *pPPO16* and *pKFDV02* in untreated leaves from seedlings of transgenic lines carrying the respective promoter in 'Golden Delicious' background. *GUS* raw expression levels for each sample are relative to the corresponding mean value of the sample *pPPO16:GUS*-nt (T0), and normalized with *ACTIN*. Numbers (1) and (2) represent independent lines of *p35S:GUS*. Bars represent SEM from 3 biological repeats (n=3). Letters indicate statistical classes (Kruskal Wallis, $p < 0.05$)
